# Retrospective Analysis of *Paenibacillus urinalis* Cultures Primarily Obtained from Sterile Sites in a Pediatric Population from Turkey

**DOI:** 10.3390/microorganisms14081661

**Published:** 2026-07-29

**Authors:** Esra Çiftci, Cüneyt Özakın, Sinem İrez Çetin, Oktay Rodoplu, Zeynep Gizem Ergün Özdel, Nazmiye Ülkü Tüzemen, Pelin Laleoğlu, Deniz Camcı Erten, Solmaz Çelebi, Mustafa Kemal Hacımustafaoğlu

**Affiliations:** 1 Department of Pediatric Infectious Diseases, Faculty of Medicine, Bursa Uludağ University, 16059 Bursa, Turkey; eciftci@uludag.edu.tr (E.Ç.); sinemirez@uludag.edu.tr (S.İ.Ç.); pelinlaleoglu@uludag.edu.tr (P.L.); solmaz@uludag.edu.tr (S.Ç.); 2 Department of Medical Microbiology, Faculty of Medicine, Bursa Uludağ University, 16059 Bursa, Turkey; ozakin@uludag.edu.tr (C.Ö.); oktayrodoplu@uludag.edu.tr (O.R.); utuzemen@uludag.edu.tr (N.Ü.T.); 3 Division of Social Pediatrics, Department of Pediatrics, Faculty of Medicine, Bursa Uludağ University, 16059 Bursa, Turkey; zeynepgizem@uludag.edu.tr; 4 Department of Pediatric Infectious Diseases, Bursa City Hospital, 16110 Bursa, Turkey; deniz.camci@hotmail.com

**Keywords:** *Paenibacillus urinalis*, pediatric, blood culture

## Abstract

In the literature, there is a limited number of case reports of *Paenibacillus urinalis* in adults. However, there is no information available regarding *P. urinalis* infection in children and infants. *Paenibacillus* growth in blood and sterile site cultures is generally considered contamination; it is unclear whether *P. urinalis* is the true cause of the infection. In this retrospective study, we aimed to clinically evaluate *P. urinalis* growth in the blood cultures of children at a tertiary referral hospital over a 5.5-year period, with identification performed using the MALDI-TOF MS method. A total of 170 hospitalized children showed *P. urinalis* growth in sterile site cultures (blood: 162; catheter: 7; CSF: 1), with forty percent of all growth occurring in only three wards (neonatal intensive care unit, hematology, oncology). Of 170 cultures, 5 (2.9%) showed further *P. urinalis* growth in control cultures, which could be considered significant. In cases with a second culture, the average growth time (time to positivity) was relatively short, at 24 + 7 h (mean + SD, min-max: 17–33 h). No mortality was observed in any case. Evaluations were conducted by the Hospital Infection Control Committee to determine the source of *P. urinalis* contamination. *P. urinalis* growth was detected in 33% of samples taken from clean, packaged linens before use; 53% of ambient air cultures; and 65% of hospitalized patient skin swabs. In conclusion, *P. urinalis* blood culture growths are primarily considered contamination; however, these growths should be carefully evaluated in risk groups (especially those admitted in neonatal intensive care units and young infants less than 3 months old). In hospitalized patients, clustered growths may be influenced by environmental (such as clean packaged linens and ambient air) and skin colonization.

## 1. Introduction

The name *Paenibacillus* comes from the Latin Paene (meaning immediately, completely) and Bacillus, meaning almost completely rod-like bacteria [1]. *Paenibacillus* was originally considered within the genus Bacillus, though was defined as a separate genus in 1993 [1,2]. *Paenibacillus* is generally a rod-like, aerobic or facultative anaerobic, endospore-forming, Gram-variable staining bacterium.

*Paenibacillus* infections in infants have been primarily identified in recent years due to advances in bacterial identification diagnostic tests. In classical methods where bacterial identification was insufficient, the *Paenibacillus* genus was identified as a Bacillus species. Matrix-Assisted Laser Desorption/Ionization Time-of-Flight Mass Spectrometry (MALDİ-TOF MS) and rRNA genetic evaluations have clarified *Paenibacillus* identification and allowed for species-level identification.

The *Paenibacillus* genus, including *P. urinalis*, is very widespread in nature (in soil, plant roots, humans, animals, environmental samples, etc.) [1] and many *Paenibacillus* species can secrete substances such as antimicrobials, pesticides, plant growth-promoting factors, and probiotics; therefore, this type of bacterium may also be important from an agricultural perspective [1,2,3]. *Paenibacillus* can contain enzymes that cause spoilage of milk and dairy products and their deterioration. In raw milk, the vast majority (>95%) of bacteria that grow during its natural shelf life in the refrigerator (e.g., over 10 days) are *Paenibacillus*. *Paenibacillus* endospores can survive in harsh environmental conditions (high heat, pressure, biocides, ultraviolet radiation, pasteurization, etc.); therefore, they can be detected in foods (plant roots and milk and dairy products, whether pasteurized or not) [4]. Their presence in environmental samples may increase the contamination risk in a hospital setting.

*Paenibacillus* generally does not contain virulence factors that can lead to invasive or opportunistic infections and that tend to occur in high-risk groups and immunocompromised individuals; therefore, its growth in sterile areas such as blood is generally considered to represent contamination. However, in children and adults, it can lead to infections in risk groups (such as prematurity, hydrocephalus, chronic kidney disease, sickle cell anemia, malignancy, and other immunosuppressive conditions), though in many of these cases, it may also not be clear whether *Paenibacillus* is the actual cause of the infection [1,2].

There are over 200 identified species of the *Paenibacillus* genus [3], over 20 of which have been identified in human clinical samples (such as *Paenibacillus thiaminolyticus*, *P. alvei*, *P. dendritiformis*, and *P. urinalis*). While species such as *P. thiaminolyticus*, *P. alvei*, and *P. dendritiformis* have been reported as infectious agents in newborns and infants, a wide variety of species have also been reported as infectious agents in adults [1,2,3,5,6,7].

The optimal treatment for *Paenibacillus* infections is unclear. *P. urinalis* is not included in the current EUCAST tables, and there are no defined clinical susceptibility breakpoints. *Paenibacillus* infections are generally resistant to penicillin, ampicillin, vancomycin (sometimes), and macrolides, though in most human cases, *Paenibacillus* isolates are considered susceptible to third-generation cephalosporins (such as ceftriaxone and cefotaxim), aminoglycosides, meropenem, and new quinolones (such as moxifloxacin and levofloxacin) [2,5,7]. In antibiograms performed for *Bacillus* spp., meropenem is considered susceptible if its minimal inhibitory concentration (MIC) value is below 0.25, ciprofloxacin if its MIC value is below 0.001, and vancomycin if its MIC value is below 2 [8,9].

In the literature, a limited number of *P. urinalis* cases in adults have been reported [5]; however, no information has been found regarding *P. urinalis* infection in children and infants. This retrospective study aimed to clinically evaluate the growth of *P. urinalis* in sterile site pediatric cultures in a tertiary reference hospital over a 5-year period.

## 2. Materials and Methods

Study design: This is a descriptive retrospective study evaluating patients under the age of 18 who were hospitalized in the Pediatric Clinics of Bursa Uludağ University (BUU) Faculty of Medicine Hospital, a tertiary referral hospital, between January 2020 and July 2025, and who showed *P. urinalis* growth in sterile site cultures. Approval for the study was obtained from the BUU Health Research Ethics Committee (ethics committee decision dated 3 December 2025 and numbered 2025/993/21-8), and it was conducted in alignment with the Helsinki Declaration.

Culturing sites and growth of *P. urinalis*: Only sterile site (blood, catheter, and CSF) culture growths from all pediatric wards were included in this study, with no urine, peritoneal or pleural culture growths. Blood samples were inoculated into BACTEC™ Peds Plus/F culture bottles (Becton, Dickinson and Company, Cockeysville, MD, USA) under aseptic conditions and incubated in the BD BACTEC™ FX200 (Becton, Dickinson and Company, Cockeysville, MD, USA) automated blood culture system until microbial growth was detected, or for a maximum of 5 days if no growth occurred. During the incubation period, the system automatically assessed the presence of growth by continuously monitoring the carbon dioxide levels resulting from microbial metabolism in the vials. After five days of incubation, passages were made from vials showing growth (positive) onto blood and eosin methylene blue agar under sterile conditions. These media were then incubated at 37 °C for 24 h under atmospheric conditions, after which the developing colonies were identified using an MALDİ-TOF MS (Bruker Daltonics, Bremen, Germany) system.

Clinical evaluation of *P. urinalis* growth: Since *P. urinalis* growth is generally considered contamination, standard culture antibiograms were not performed. A second culture was taken from cases where culture growth was reported, and in cases with *P. urinalis* growth, simultaneous growth of other bacteria was also recorded. The culturing time (time to positivity; TTP) of the first (N = 170) and second positive cases (N = 5) was extracted from the records.

*P. urinalis* source investigation: To determine the source of *P. urinalis* infection, assessments and evaluations were conducted twice, one year apart, by the Hospital Infection Control Committee. For this purpose, environmental cultures were taken from different pediatric wards (in July 2024 and July 2025). Within this framework, skin swab and environmental cultures, sterile syringe, and antiseptic solution culture samples were taken from hospitalized patients in clinics where growth was observed and evaluated.

Statistical methods: Descriptive statistics were used to analyze *P. urinalis* growth rates based on the admission ward and patient age. To evaluate potential differences in TTP between the initial positive cultures and subsequent control cultures obtained from the same patient at different time points, paired comparisons were performed. Given the small sample size and inability to robustly assume normality, the nonparametric Wilcoxon signed-rank test was employed instead of the parametric paired-samples *t*-test, with a *p*-value of less than 0.05 considered statistically significant. All analyses were conducted using IBM Corp. (2013), IBM SPSS Statistics for Windows, Version 22.0, Armonk, NY, USA: IBM Corp.

## 3. Results

Over a five-year period, a total of 170 hospitalized children showed *P. urinalis* growth in sterile area cultures (blood: 162; catheter: 7; CSF: 1) (Table 1).

The average age of the patients was 45,47 ± 57,67 months (Mean ± SD, min-max: 0–202 months; median: 17 months). Of all patients, 52.3% (*n* = 89) were boys and 47.6% (*n* = 81) were girls. Additionally, one case (13 days old) showed growth in a conjunctival culture, which was not included in this study because of this being a non-sterile site culture. The distributions of cases with *P. urinalis* growth according to age groups and wards and sterile site cultures according to years and services are presented in Table 1, Table 2 and Table 3.

More than one-third of the cases (34.1%) were ≤3 months old, and more than half (51.1%) were <2 years old. More than a quarter of the cases (28.8%) were detected in the neonatal intensive care unit, and 40% of all *P. urinalis* growths occurred in only three wards (neonatal intensive care unit, hematology, oncology) (Table 2). Except for one (CSF) case, all growths were blood-derived (95.2% blood, 4.1% catheter). No simultaneous blood culture growth (*P. urinalis*) was detected in the catheter cultures. In seven of the cases with initial *P. urinalis* growth (7/170; 4.1%), simultaneous co-growths (three *Staphylococcus capitis*, one *Staphylococcus hominis*, one *Staphylococcus haemolyticus*, one *Staphylococcus cohni*, one *Corynebacterium striatum*) were detected. In 5 of the 170 cultures (2.9%), *P. urinalis* growth was again detected in the control culture. Five cases second growth cases were considered significant, and antibiotic treatment was reevaluated; however, no clinical deteriorations were seen in these patients. Their clinical data are presented in Table 4. In these cases, no accompanying co-growth was detected in the culture, and in four of the five, *P. urinalis* growth was not detected in the third control culture, while *Streptococcus mitis* grew in one case. One case was discharged before the third culture could be taken.

In those with recurrent growth (five cases), the mean TTP in the first *P. urinalis* growth case culture was 28 ± 18 h (mean ± SD, min-max: 12–58 h), while in those with a second growth, it was 24 ± 7 h (mean ± SD, min-max: 17–33 h) (*p* = 0.4).

Before/during the first culture, 91.1% (155/170) of the cases were taking antibiotics, of which 126 (74.1%) were taking antibiotics expected to be effective against *Paenibacilli* (such as cefotaxime, ceftriaxone, cefepim, meropenem, and ciprofloxacin). Fifteen (8.8%) patients were not taking antibiotics during the first culture growth period. and no growth was detected in the control culture of any of these patients. An antibiogram was not performed because *P. urinalis* growth was reported as contamination. The initial *P. urinalis* growth was principally not considered an indication for changing patient treatment; however, in five cases where a second growth occurred which could be clinically significant, the treatments were evaluated to cover effective antibiotics such as cefotaxim or meropenem, taking into account findings from the literature. No patients were lost.

The 23-day-old infant with CSF growth had clinical findings consistent with sepsis/meningitis, with 46 mg/dL glucose, 109 mg/dL protein, rare leukocytes, and no bacteria on direct Gram staining.

The environmental culture results from different pediatric wards are shown in Table 5. In this context, *P. urinalis* growth was detected in 33% (15/45) of samples taken from clean, packaged linens before use from different pediatric wards (neonatal and pediatric intensive care units, pediatric hematology, pediatric oncology, pediatric health clinic, laundry, etc.). In ambient air cultures (neonatal intensive care unit, pediatric clinic, laundry, operating room, etc.), *P. urinalis* growth was observed in 53.8% (7/13) of air samples. On the other hand, *P. urinalis* was not detected in any of the environmental room samples taken from four different wards (neonatal and pediatric intensive care units, pediatric hematology, pediatric oncology). No growth was detected in a total of 15 different sterile syringes in the neonatal intensive care unit, pediatric oncology, and pediatric clinics; in detergents and additives used in laundry; or in antiseptics containing alcohol, iodine, and chlorhexidine used in the area. Of 20 skin swab cultures taken from 10 patients in the neonatal intensive care unit and pediatric clinics, 65% (13/20) showed *P. urinalis* growth. While 60% of swabs were positive after disinfection with 2% chlorhexidine/70% isopropyl alcohol before collection, growth was detected in 70% (7/10) of swab samples taken without disinfection.

## 4. Discussion

### 4.1. General Clinical Features and Framework of Paenibacillus Infections Other than P. urinalis

*Paenibacillus* spp. growth in sterile site cultures may indicate contamination; however, it is known that many non-invasive bacterial agents can be the actual cause of infection in risk groups and immunocompromised individuals. *Paenibacillus*, particularly *P. thiaminolyticus*, can cause sepsis and meningitis in newborns and can lead to adverse neurological sequelae, including mortality [1,2,6,7]. In a prospective study of term neonatal sepsis in Uganda, PCR detected *P. thiaminolyticus* (70%) or *Paenibacillus* spp. (30%) in 6% (37/631) of clinical neonatal sepsis cases, 30% of which showed poor prognosis, and 14% of these cases died within the first year. Approximately 20% of survivors had neurological sequelae, including post-infectious hydrocephalus [7]. However, there are currently no data reported in the literature that indicate that *P. urinalis* is a serious clinical infection agent in children (including neonates).

In a recent large-scale study, 177 *Paenibacillus* infections in children and adults were evaluated across 40 articles/reports [2], with a total of 38 infections in adults caused by 23 *Paenibacillus* species identified in the literature review. Adult infections were generally seen in risk groups, the clinical findings varied, and the prognosis was generally good (only two mortalities, approximately 4%). No particular *Paenibacillus* type was observed in adults [2]. Infected infants generally presented with sepsis, meningitis, and hydrocephalus; almost all cases in infants developed before the age of 3 months. Among infant infections, some types such as *P. thiaminolyticus* (79%), *P. alvei* (1%), and *P. dendritiformis* (1%) were particularly prevalent, along with other species that could not be distinguished but were only detected at the genus level (19%). All newborns and young infants with *Paenibacillus* infection presented with severe indicators of infection (meningitis, sepsis, hydrocephalus, severe cerebral destruction, etc.) and had a high mortality rate (17%) [2]. The authors’ interpretation is that *Paenibacillus* infections in newborns and infants have a different etiology and a worse prognosis than in adults (2). Furthermore, these data suggest that some *Paenibacillus* species may present with different clinical manifestations and carry a higher risk of being more invasive, depending on the age group, though these reports do not include any specific clinical notifications regarding *P. urinalis* infections.

### 4.2. Clinical Evaluation and Significance of Cases with P. urinalis Growth

In the literature review, data on *P. urinalis* infections are very limited. It was first isolated and identified in 2008 from the urine of a woman without signs of urinary tract infection [8]. In a tertiary hospital in Korea, *P. urinalis* growth was detected in blood cultures of adults aged 21–76 years, who were generally in a risk group (osteosarcoma, intracerebral hemorrhage, sinusitis treated with acupuncture, subarachnoid hemorrhage, aspiration pneumonia, etc.). With antibiotic treatment (such as moxifloxacin, levofloxacin, ceftriaxone, and piperacillin–tazobactam), fever subsided within days and clinical improvement was observed. The authors considered most cases to represent contamination [5], and no cases of clinical *P. urinalis* infection in children were found in the literature review.

Generally, for sterile site bacterial growth, the presence of positive blood and CSF cultures generally provides evidence to determine the etiology. Even if blood cultures are positive, in some cases, agents thought to be non-invasive may be considered as contamination; however, in risk groups and immunocompromised individuals, the determination of contamination should be made with caution, as positive double-arm blood cultures and/or simultaneous growth or early growth signals (TTP: <24–48 h) in peripheral blood catheter samples suggest a true infectious agent rather than contamination.

The vast majority of our cases were hospitalized at-risk infants and children usually with many co-morbidities (such as neutropenic, immune-suppressed, and hematopetic stem cell or solid organ transplant patients; neonatal intensive care unit patients with very/extremely low birth weight or very small preterms; intraventicular hemorrhage; requiring different operations, etc.), so many received combined antibiotics for their diseases. We did not intend to change the antibiotics based on the initial *P. urinalis* results, because *P. urinalis* was considered as contamination. However, during the second growth, although there was no obvious clinical deterioration, we also evaluated the antibiotics empirically to cover *P. urinalis*.

Since no antibiograms were performed in any of our cases, regarding the EUCAST recommendations, we could not determine susceptibility; however, the administration or continuation of antibiotics reported to be potentially susceptible (such as cefotaxim/ceftriaxone and meropenem) after the second culture may explain the absence of growth in the third cultures.

There are currently no species-specific EUCAST clinical susceptibility breakpoints for *P. urinalis*, with this organism generally not listed in the breakpoint tables [9]. For *Paenibacillus* species, susceptibility testing is usually performed by broth microdilution MIC methods, or, sometimes, interpreted using the breakpoints of similar bacteria (such as *Bacillus* spp.). However, in the five cases in this study where the same agent grew a second time, despite the absence of an antibiogram, it was considered a possible agent, and regarding the literature recommendations, the empirically susceptible considered antibiotics (such as cefotaxim/ceftriaxone, meropenem, aminoglycoside, and quinolones) were continued. In cases with a second bacterial growth (approximately 3%), the TTP was found to be an average of 24 h (17–33 h). No patient experienced a third bacterial growth, and no mortality was observed.

Similarly, in one of our cases where CNS (central nervous system) infection was suspected and pleocytosis was detected in the CSF, bacterial growth may have indicated a possible infection, but had a good prognosis.

In our opinion, the *P. urinalis*-positive cultures did not affect the patients’ condition, so specific antibiotic change was not required. The second instance of positivity for the same culture (and even with a relatively short TTP) in five patients can still be considered contamination again. True blood culture positivity with low *P. urinalis* virulence may cause an infection, but without obvious clinical findings (and might be cleared without specific treatment due to lacking virulence/invasive genes) (Table 4). The improvement and absence of a negative outcome in one case, who was discharged without treatment, may also support the possibility of a second contamination, or non-virulent bacteremia resolving spontaneously. In this context, it can be said that even if *P. urinalis* is the causative agent of an infection, it is not invasive and has a good prognosis.

Although *P. urinalis* is named as such because it was first isolated from the urine of a female patient [8], there was no urine positivity found in our patients. Also, there were no peritoneal or pleural yielding.

### 4.3. Investigations Regarding Paenibacillus and P. urinalis Source

Studies of sources of *Paenibacillus* growth other than *P. urinalis*: In *Paenibacillus* infections generally, the source of the causative agent may not be clear. In one study, it was suggested that the agent may originate from non-sterile practices involving the umbilical cord of newborns [7]. One study in the US noted that neonatal *Paenibacillus* infections were seen as a risk factor in premature babies or babies of mothers using narcotics [2]. Furthermore, it has been suggested that preparations containing *Paenibacillus* as probiotics should not be used in newborns until safety is proven [2]. In a study conducted in Uganda, *Paenibacillus* was not detected by PCR in maternal blood or in vaginal, placental, and cord blood samples of infants who developed *Paenibacillus* infection, suggesting that neonatal *Paenibacillus* infections may not be maternally related [6].

Investigation of *P. urinalis* growth source in our study: The common presence of *P urinalis* in environmental samples can increase the risk of contamination in a hospital setting. In our study, under the guidance of the Hospital Infection Control Committee, two-stage environmental and screening cultures were obtained to investigate the source of possible *P. urinalis* contamination (Table 5). *P. urinalis* growth was not detected in sterile syringes, antiseptics (such as alcohol, iodine, and chlorhexidine), detergents used in room cleaning and laundry, or environmental samples taken from rooms. On the contrary, *P. urinalis* growth was found in 33% of clean packaged sheet samples, 53% of room air samples, and 65% of skin swab cultures from hospitalized patients, suggesting that the primary factor in blood culture growth may be due to contaminated clean linens and associated airborne transmission, which can result from the survival capacity of *Paenibacillus* in harsh conditions.

The fact that more than half of the skin swab cultures from hospitalized patients showed growth supports the possibility of blood culture contamination via skin contact. Pre-treatment of skin swabs with 2% chlorhexidine/70% isopropyl alcohol (60% growth) did not significantly affect *P. urinalis* growth compared to no treatment (70% growth), supporting the idea that blood cultures can be contaminated despite chlorhexidine/alcohol disinfection due to skin colonization. *P. urinalis* is not considered among the commensal bacteria of the skin; however, its detection at high levels in our cases (even after routine skin disinfection) suggests that *P. urinalis* is a bacterium that can easily adapt to the skin. We do not know whether skin colonization caused by linen or airborne contamination is secondary and/or transient and did not evaluate the colonization duration.

Study limitations: Important limitations of this study include its single-center, retrospective nature and the fact that no antibiogram was performed. In cases with a second culture, although the same pathogen recurred and the relatively shorter TTP supported true growth, in actuality, there was no clear proof of these five patients having true infection. We did not perform outbreak analysis of these patients (especially for the last three who clustered in the same month), which may be another limitation of our study.

Study strengths: This is, to our knowledge, the largest study conducted only in pediatric clinics evaluating positive blood culture growth of *P. urinalis*. The distribution of cases according to age group and ward and the average TTP time determination can be considered advantages of this study. In the source analysis, evaluating/demonstrating the causes of blood contamination (such as the correlation between the detection of the pathogen in bed linens and room air, consistent with the characteristics of *P. urinalis*) can contribute to the literature in terms of preventing possible contaminations. Additionally, the fact that *P. urinalis* is frequently detected on the skin of hospitalized patients (not previously mentioned), despite not being a commensal bacterium, and its possible long-term presence/adaption to the skin flora, can be considered among the strengths of this study in terms of contributing to the literature.

## 5. Conclusions

*P. urinalis* blood culture growth is primarily considered a form of contamination. Even if, in the case of true infection, the clinical outcome may be mild due to lacking virulence factors. However, these growths should be carefully evaluated in high-risk groups (especially in infants under 3 months of age and those admitted to the NICU). In clustered growths in hospitalized patients, environmental (such as clean packaged linens and ambient air) and skin colonization may play a role, so preventive measurements should be taken while considering *P. urinalis* specialties.

## Figures and Tables

**Table 1:** *P. urinalis* growth by age.

	Blood Culture	Catheter Culture	CSF Culture	Total
≤3 months	58 (34.1%)	3 (1.76%)	1 (0.58%)	62 (36.4%)
4–23 months	30 (17.6%)	1 (0.58%)	-	31 (18.2%)
24–83 months	35 (20.5%)	2 (1.2%)	-	37 (21.7%)
≥84 months	39 (22.9%)	1 (0.58%)	-	40 (23.5%)
Total	162 (95.2%)	7 (4.1%)	1 (0.58%)	170 (100%)

**Table 2:** *P. urinalis* distribution according to ward of admission.

	Blood Culture	Catheter Culture	CSF Culture	Total
NICU	49 (28.8%)	2 (1.1%)	1 (0.58%)	52 (30.5%)
PICU	6 (3.5%)	2 (1.1%)	-	8 (4.7%)
Ped Hematology	7 (4.1%)	2 (1.1%)	-	9 (5.2%)
Ped Oncology	13 (7.6%)	1 (0.58%)	-	14 (8.2%)
Other wards	87(51.1%)	-	-	87 (51.1%)
Total	162 (95.2%)	7 (4.1%)	1 (0.58%)	170 (100%)

Abbreviations: NICU; neonatal intensive care unit, PICU; pediatric intensive care unit, Ped; Pediatric, CSF; cerebrospinal fluid.

**Table 3:** *P. urinalis* sterile site cultures according to years and services.

Pediatric Wards	2020	2021	2022	2023	2024	2025	Total N (%)
NICU	-	4	8	13	24	3	52 (30.5)
PICU	1	2	1	2	1	1	8 (4.7)
Pediatric Infectious Diseases	4	2	5	6	7		24 (14.1)
Pediatric Clinic-1 *	4	7	12	10	15	1	49 (28.8)
Pediatric Clinic-2 **			3	1			4 (2.3)
Pediatric hematology			1	3	4	1	9 (5.2)
Pediatric oncology	1	1		5	6	1	14 (8.2)
Pediatric Surgery	1			3	4	2	10 (5.9)
Total	11	16	30	43	61	9	170 (100)

Abbreviations: NICU; neonatal intensive care unit, PICU; pediatric intensive care unit. * Pediatric Clinic 1: Pediatric Nephrology, Pediatric Neurology, Pediatric Gastroenterology, Pediatric Immunology, Pediatric Rheumatology. ** Pediatric Clinic 2: Pediatric Metabolism, Pediatric Cardiology, Pediatric Allergy, Pediatric Endocrinology.

**Table 4:** Some clinical features of 5 patients having second *P. urinalis* culture positivity.

Patient	Age/Month	Diagnosis	Treatment During the First Culture	Treatment Change After the 1st Culture Results Available *	Treatment Change After the 2nd Culture Results Available **	3rd Culture	Prognosis
1, MY	152	Mesenteric lymphadenopathy Sepsis	Cefotaxime, Metronidazole	No change	No change	No culture growth	survive
2, ME	1	Late neonatal sepsis, Hypoglycemic convulsion	Vancomycin, Gentamicin, TMP-SMX Ciprofloxacin	Fluconazole has been added	(No change) Vancomycin, Gentamicin, Ciprofloxacin, Fluconazole	No culture growth	survive
3, EG	0 (15 d)	Late neonatal sepsis, Hypoxic ischemic encephalopathy	Vancomycin Piperacillin/ tazobactam Amikacin	Piperacillin-tazobactam stopped, meropenem has been added	(No change) Vancomycin, Meropenem, Amikacin	No culture growth	survive
4, İA	14	Persisted convulsions Sepsis	Ampicillin/Sulbactam	No change	No change	Not taken ***	survive
5, PY	0 (1 d)	Prematurity (35 gw) Late neonatal sepsis	Ampicillin Gentamicin	Cefotaxime Amikacin	(No change) Cefotaxime Amikacin	*Streptococcus mitis*	survive

Patients 2, 3 and 5 were from NICU, with multiple co-morbidities. The time of 2nd cultures were 8 January 2023, 8 July 2023, 17 June 2024, 19 June 2024, and 30 June 2024, for patients 1 to 5, respectively. *: Treatment change after the 1st culture results available, were principally made empirically (mainly in regard of general clinical and laboratory findings, as well as assuming effective for *P. urinalis*). **: No treatment change were made after the 2nd culture results available, because of no clinical deterioration, and assuming the antibiotics given will be effective for *P. urinalis*. ***: The 2nd culture result had been available after the discharge of the patient, The patient was well in the control. So, 3rd culture was not taken.

**Table 5:** *P. urinalis* environmental cultures.

Environmental Culture Sites *	*P. urinalis* Positivity n/N (%)
Clean packaged linens before use, N = 45	15/45 (33)
Ambient air cultures N = 13	7/13 (54)
Sterile syringes, N = 15	0/15 (0)
Room environmental samples, N = 6	0/6 (0)
Antiseptics (70% alcohol, 10% povidone-iodine, 2% chlorhexidine), N = 5	0/5 (0)
Detergents for room cleaning, N = 4	0/4 (0)
Skin swab cultures, N = 10 patients, 20 times(including the both sides)	13/20 (65)
Before disinfection ** (left side), N = 10	7/10 (70)
After disinfection ** (right side), N = 10	6/10 (60)

*: Environmental cultures were taken from neonatal intensive care unit, pediatric intensive care unit, hemato-oncology, other pediatric awards, operating rooms, laundry rooms. Skin swap cultures were taken from the patients staying in NICU, PICU, hemato-oncology wards. **: 2% clorhexidine/70% isopropyl alcohol.

## Data Availability

The data presented in this study are available on request from the corresponding author.
