# Correction: Çiftci et al. Retrospective Analysis of *Paenibacillus urinalis* Cultures Primarily Obtained from Sterile Sites in a Pediatric Population from Turkey. *Microorganisms* 2026, *14*, 1661

**DOI:** 10.3390/microorganisms14091955

**Published:** 2026-09-04

**Authors:** Esra Çiftci, Cüneyt Özakın, Sinem İrez Çetin, Oktay Rodoplu, Zeynep Gizem Ergün Özdel, Nazmiye Ülkü Tüzemen, Pelin Laleoğlu, Deniz Camcı Erten, Solmaz Çelebi, Mustafa Kemal Hacımustafaoğlu

**Affiliations:** 1 Department of Pediatric Infectious Diseases, Faculty of Medicine, Bursa Uludağ University, 16059 Bursa, Turkey; eciftci@uludag.edu.tr (E.Ç.); sinemirez@uludag.edu.tr (S.İ.Ç.); pelinlaleoglu@uludag.edu.tr (P.L.); solmaz@uludag.edu.tr (S.Ç.); 2 Department of Medical Microbiology, Faculty of Medicine, Bursa Uludağ University, 16059 Bursa, Turkey; ozakin@uludag.edu.tr (C.Ö.); oktayrodoplu@uludag.edu.tr (O.R.); utuzemen@uludag.edu.tr (N.Ü.T.); 3 Division of Social Pediatrics, Department of Pediatrics, Faculty of Medicine, Bursa Uludağ University, 16059 Bursa, Turkey; zeynepgizem@uludag.edu.tr; 4 Department of Pediatric Infectious Diseases, Bursa City Hospital, 16110 Bursa, Turkey; deniz.camci@hotmail.com

In the original publication [1], Table S1 was not cited. The citation has now been inserted in Section 2, Paragraph 1 and should read:

Study design: This is a descriptive retrospective study evaluating patients under the age of 18 who were hospitalized in the Pediatric Clinics of Bursa Uludağ University (BUU) Faculty of Medicine Hospital, a tertiary referral hospital, between January 2020 and July 2025, and who showed *P. urinalis* growth in sterile site cultures (Table S1). Approval for the study was obtained from the BUU Health Research Ethics Committee (ethics committee decision dated 3 December 2025 and numbered 2025/993/21-8), and it was conducted in alignment with the Helsinki Declaration.

A correction has also been made to the Supplementary Materials:

**Supplementary Materials:** The following supporting information can be downloaded at: https://www.mdpi.com/article/10.3390/microorganisms14081661/s1. Table S1: *P. urinalis* culture growths of our pediatric patients.

The authors state that the scientific conclusions are unaffected. This correction was approved by the Academic Editor. The original publication has also been updated.
